# SiH/TiO_2_ and GeH/TiO_2_ Heterojunctions: Promising TiO_2_-based Photocatalysts under Visible Light

**DOI:** 10.1038/srep04810

**Published:** 2014-05-02

**Authors:** Mang Niu, Daojian Cheng, Dapeng Cao

**Affiliations:** 1 State Key Laboratory of Organic-Inorganic Composites, Beijing University of Chemical Technology, Beijing 100029, P. R. China

## Abstract

We use hybrid density functional calculations to find that the monolayer silicane (SiH) and the anatase TiO_2_(101) composite (*i.e.* the SiH/TiO_2_ heterojunction) is a promising TiO_2_-based photocatalyst under visible light. The band gap of the SiH/TiO_2_(101) heterojunction is 2.082 eV, which is an ideal material for the visible-light photoexcitation of electron-hole pairs. Furthermore, the SiH/TiO_2_(101) heterojunction has a favorable type-II band alignment and thus the photoexcited electron can be injected to the conduction band of anatase TiO_2_ from that of silicane. Finally, the proper interface charge distribution facilitates the carrier separation in the SiH/TiO_2_(101) interface region. The electron injection and carrier separation can prevent the recombination of electron-hole pairs. Our calculation results suggest that such electronic structure of SiH/TiO_2_(101) heterojunction has significant advantages over these of doped TiO_2_ systems for visible-light photocatalysis.

Since the discovery of photoinduced decomposition water on TiO_2_ electrodes was reported by Fujishima and Honda in 1972^1^, TiO_2_ has been considered as one of the most promising photocatalytic materials for solar energy conversion and environmental purification^2^,^3^,^4^ due to its low cost, high stability, and long life-time of photogenerated carrier. However, its large band gap (3.0 eV for rutile and 3.2 eV for anatase) absorbs only the ultraviolet light (about 4% of the sunlight) for the photoexcitation of electron-hole pairs, thus leading to the low photocatalytic activity under visible-light irradiation.

In the past decades, great efforts have been devoted to extending the photocatalytic activity of TiO_2_ to the visible-light range. It has been reported that doping (or codoping) modification is the most common approach to modify the band gap and enhance the photocatalytic activity of TiO_2_^5^,^6^,^7^,^8^,^9^,^10^,^11^,^12^,^13^. Unfortunately, the practical application of heteroatom-doped TiO_2_ is restricted by the high recombination rate of electron-hole pairs, the strongly localized impurity states, and the low stability against photocorrosion^14^,^15^,^16^,^17^. Recently, the composites of TiO_2_ and other semiconductor materials have been proposed to overcome the shortcomings of doping^18^,^19^,^20^,^21^. For example, Zhou et al.^19^ demonstrated that the MoS_2_ nanosheet-coated TiO_2_ nanobelt exhibits high performance of photocatalytic hydrogen production and organic dye degradation, in which the matched band energy favors the charge transfer and suppresses the recombination of electron-hole pairs between MoS_2_ and TiO_2_. In addition, Liang and coworkers^20^ reported that the carbon nanotube (CNT) acts as photosensitizer in CNT/TiO_2_ composites, leading to great enhancement of CO_2_ photoreduction under visible visible-light irradiation. However, it is still necessary to explore new TiO_2_-based photocatalysts under visible light in order to enable their wider applications in future.

After the discovery of honeycomb monolayers of group IV elements (graphene, silicene, and germanene), the research on the band gap opening and controlling has experienced a rapid growth for the potential applications of these materials^22^,^23^,^24^,^25^. One of the effective ways to open the band gaps of graphene, silicene, and germanene is hydrogenation. For example, Bianco et al.^25^ found that the hydrogenation of germanene can produce the germanane (GeH) with a direct band gap of 1.55 eV, which has great potential for optoelectronic and sensing applications. In addition, the graphane (CH) is an insulator with band gap larger than 3.5 eV^23^ and the electronic properties of silicane (SiH) has not been reported in previous experiments. As mentioned above, the small band gap semiconductors (such as MoS_2_ and CNT) can play an important role in the photosensitization of TiO_2_. Therefore, it is very interesting to study silicane and germanane as photosensitizers to enhance the visible-light photocatalysis of TiO_2_. Unfortunately, both experimental and theoretical investigations on this issue are still scarce.

In this work, we choose silicane and germanane as photosensitizers on the anatase TiO_2_(101) surface and investigate the electronic properties of SiH/TiO_2_(101) and GeH/TiO_2_(101) heterojunctions by using hybrid density functional calculations. It is found that the monolayer silicane sensitization is a promising way to modify the electronic structures of anatase TiO_2_ for enhancing visible-light photocatalysis. Our calculation results suggest that the SiH/TiO_2_(101) heterojunction could be a promising TiO_2_-based photocatalyst under visible light.

## Results

**Figure 1:**
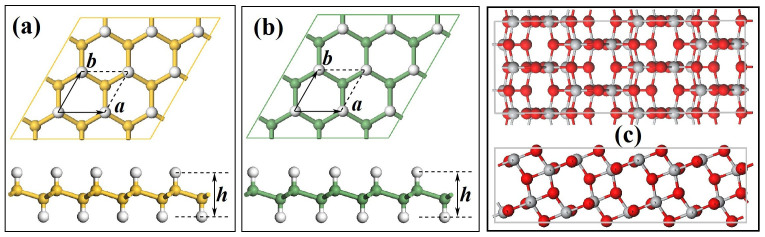
Structures of monolayer silicane, germanane, and two-layer anatase TiO_2_(101) surface. The top and side views of monolayer (a) silicane, (b) germanane, and (c) two-layer anatase TiO_2_(101) slabs, respectively, *a* and *b* are the lattice constants and *h* represents the height of monolayer silicane and germanane. The yellow, green, white, grey, and red balls represent Si, Ge, H, Ti, and O atoms, respectively.

The chair-like monolayer silicane and germanane are graphene-like hexagonal sheets with the hydrogen atoms alternating on both sides of the Si and Ge planes, as shown in Figure 1(a) and Figure 1(b), respectively. The optimized lattice parameters are *a* = 3.888 Å, *h* = 3.721 Å and *a* = 4.085 Å, *h* = 3.859 Å for monolayer silicane and germanane, respectively. Here, we choose anatase TiO_2_(101) surface as substrate to support monolayer silicane and germanane because the TiO_2_(101) surface is the most stable surface among the low index surfaces of anatase TiO_2_. More importantly, a 2 × 2 unit cell of anatase TiO_2_(101) has a rectangular cell of 20.820 Å × 7.641 Å, which is nicely matched with a 5 × 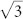
rectangular unit cell of monolayer silicane or germanane. The SiH/TiO_2_(101) and GeH/TiO_2_(101) heterojunctions were modeled by placing the monolayer silicane and germanane sheets on the top of two-layer anatase TiO_2_(101) slabs, respectively. The structure of 2 × 2 unit cell of two-layer anatase TiO_2_(101) slab is displayed in Figure 1(c). Such a slab model has been used often for theoretical calculations^21^,^26^,^27^. A vacuum region of 20 Å above TiO_2_(101) slabs in SiH/TiO_2_(101) and GeH/TiO_2_(101) heterojunctions was used to minimize the interactions between neighboring systems.

**Figure 2:**
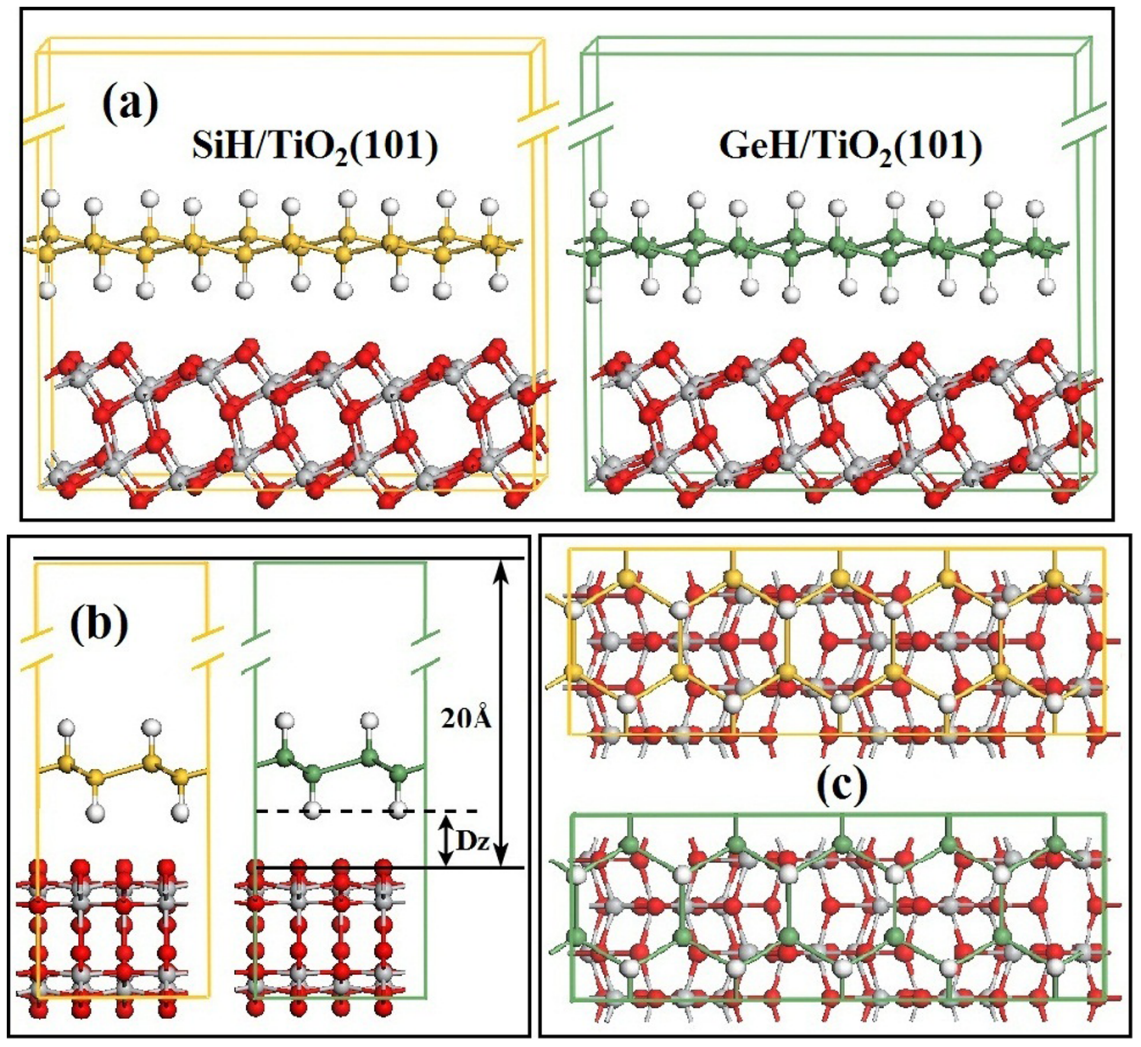
Optimized structures of SiH/TiO_2_(101) and GeH/TiO_2_(101) heterojunctions. The (a) front, (b) side, and (c) top views of optimized SiH/TiO_2_(101) and GeH/TiO_2_(101) heterojunctions, respectively, D_z_ is the distance between silicane (or germanane) and anatase TiO_2_(101) slabs.

The optimized geometries of SiH/TiO_2_(101) and GeH/TiO_2_(101) heterojunctions were illustrated in Figure 2. It is found that the silicane sheet, germanane sheet, and TiO_2_(101) slabs keep the original symmetries without obvious distortions after the formations of interfaces. The optimized surface lattices, binding energies and absorption distances of SiH/TiO_2_(101) and GeH/TiO_2_(101) heterojunctions were listed in Table 1. The values of binding energies are positive for both SiH/TiO_2_(101) and GeH/TiO_2_(101) heterojunctions, suggesting that the monolayer silicane and germanane interfaced TiO_2_(101) slabs form stable heterojunctions. In addition, it is found the absorption of germanane on TiO_2_(101) surface is stronger than that of silicane on TiO_2_(101) surface owing to the large binding energy of 0.112 eV and short absorption distance of 2.489 Å.

**Figure 3:**
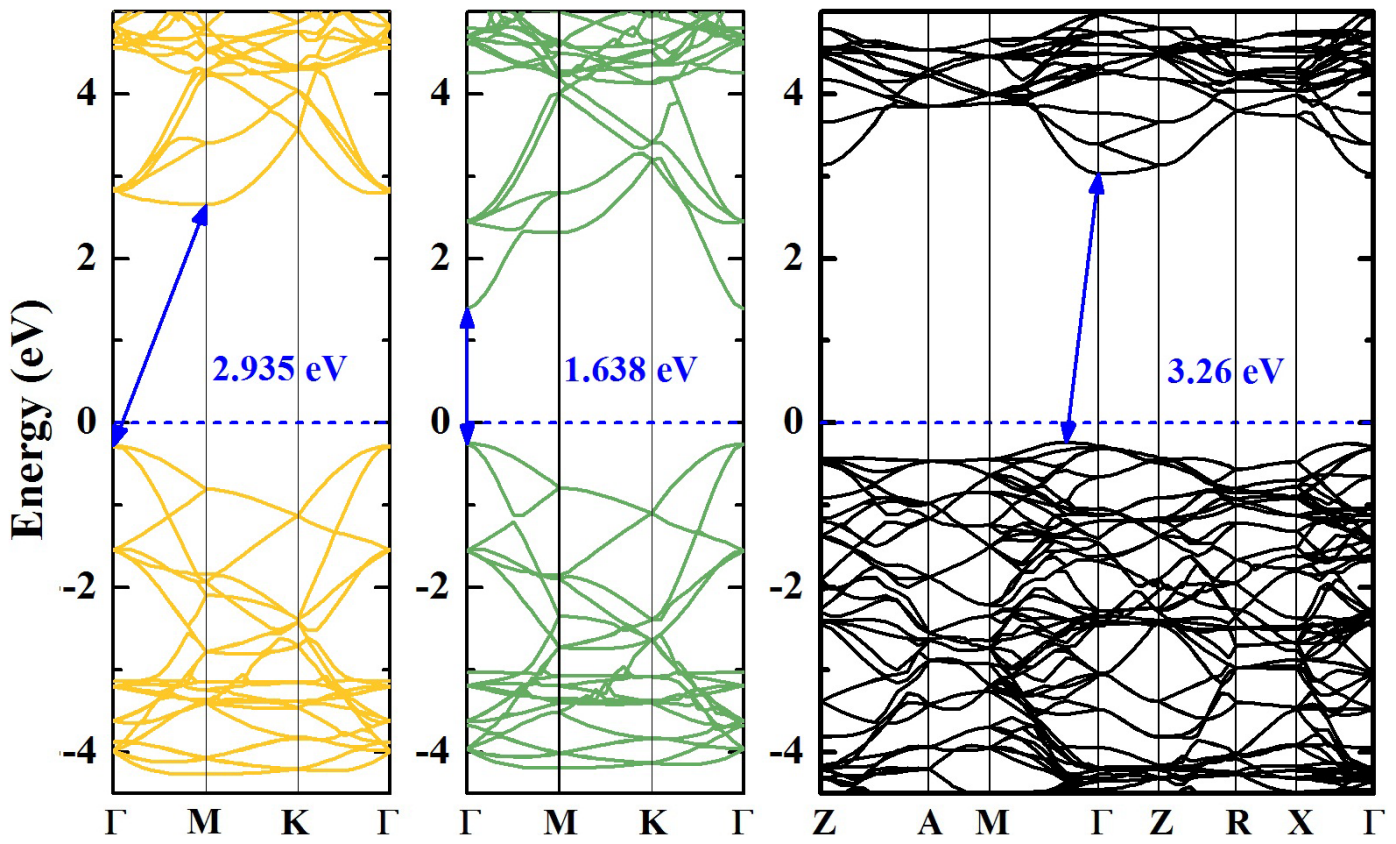
Band structures of monolayer silicane, germanane, and bulk anatase TiO_2_. HSE06 calculated band structures of monolayer silicane, germanane, and bulk anatase TiO_2_ along high symmetry lines of Brillouin zone. The band gaps are illustrated with blue lines with two arrows and the values are also displayed in blue numbers. The engery zero is taken as the Fermi level and displayed with a blue dashed line.

In order to understand the prospective photocatalytic performance of SiH/TiO_2_(101) and GeH/TiO_2_(101) heterojunctions, we first calculated the electronic structures of monolayer silicane, monolayer germanane, and bulk anatase TiO_2_ by using Heyd-Scuseria-Ernzerhof (HSE06)^28^,^29^ hybrid functional, as displayed in Figure 3. It is found that the monolayer silicane has an indirect band gap of 2.935 eV, the monolayer germanane has a direct band gap of 1.638 eV, and the bulk anatase TiO_2_ has an indirect band gap of 3.26 eV. For germanane and anatase TiO_2_, the HSE06 calculated band gaps are precisely consistent with the experimental values of 1.59 eV and 3.2 eV, respectively^25^. These results confirmed that the monolayer silicane and germanane can be used for the sensitization of antase TiO_2_ due to the narrower band gaps than that of anatase TiO_2_.

**Figure 4:**
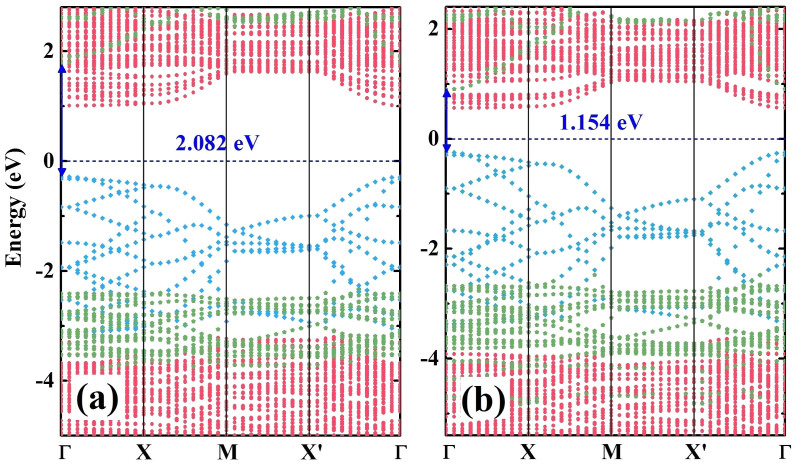
Band structures of SiH/TiO_2_(101) and GeH/TiO_2_(101) heterojunctions. HSE06 calculated band structures of (a) SiH/TiO_2_(101) and (b) GeH/TiO_2_(101) heterojunctions along high symmetry lines of Brillouin zone. The red, blue, and green dots represent the energy bands of TiO_2_, silicane (or germanane), and SiH-TiO_2_ (or GeH-TiO_2_)hybrids, respectively. The band gaps of silicane and germanane in SiH/TiO_2_(101) and GeH/TiO_2_(101) heterojunctions are illustrated with blue lines with two arrows and the values are also displayed in blue numbers. The energy zero is taken as the Fermi level and displayed with a blue dashed line.

The HSE06 calculated band structures of the SiH/TiO_2_(101) and GeH/TiO_2_(101) heterojunctions were illustrated in Figure 4(a) and Figure 4(b), respectively. The energy bands of SiH/TiO_2_(101) and GeH/TiO_2_(101) heterojunctions were projected on each atoms to identify the energy bands of monolayer sheet (silicane and germanane), TiO_2_, and sheet-TiO_2_ hybrids. It is found that energy bands of SiH/TiO_2_(101) and GeH/TiO_2_(101) heterojunctions show a typical type-II band alignments, in which the conduction band (CB) of silicane and germanane locates at higher energy states than that of TiO_2_(101) surface. The build-in potential is defined as the conduction band minimum (CBM) offset between silicane (or germanane) and TiO_2_(101) surface, which are 0.787 eV and 0.443 eV for SiH/TiO_2_(101) and GeH/TiO_2_(101) heterojunctions, respectively. After the formation of an interface, the band gaps of silicane and germanane reduced to 2.082 eV and 1.154 eV, respectively. In SiH/TiO_2_(101) and GeH/TiO_2_(101) heterojunctions, the electron can be excited from the valence band (VB) of silicane (or germanane) to its CB under visible light irradiation, leaving a hole in its VB. Then the photoexcited electron can be injected into the CB of TiO_2_ under the driving of build-in potential. Because the CB energy bands of silicane and germanane are hybridized significantly with that of TiO_2_, the injection of photoexcited electron can be transferred adiabatically^17^. After the electron injection, the photogenerated hole tends to locate at the VBM of silicane (or germanane), which is the low energy state for the hole. It is found that the interfaces of SiH/TiO_2_(101) and GeH/TiO_2_(101) heterojunctions favor the electron-hole separation so that the electron tends to locate at the CBM of TiO_2_ while the hole is located at the VBM of silicane or germanane. Therefore, the oxidation and reduction reactions can take place in silicane (or germanane) and TiO_2_, respectively. Our theortical results suggest that the visible-light photocatalytic response of anatase TiO_2_ can be improved through silicane and germanane sensitizations owing to the enhancement of visible-light absorption and the suppression of recombination of electron-hole pairs. Therefore, this small band gap semiconductor sensitization approach is better than the conventional doping or codoping methods. Considering the band gaps of silicane and germanane in SiH/TiO_2_(101) and GeH/TiO_2_(101) heterojunctions are 2.082 eV and 1.154 eV, respectively, we predicted that the SiH/TiO_2_(101) heterojunction is a promising photocatalyst for visible-light photocatalysis and the GeH/TiO_2_(101) heterojunction is a prospective photovoltaic material for solar energy conversion.

**Figure 5:**
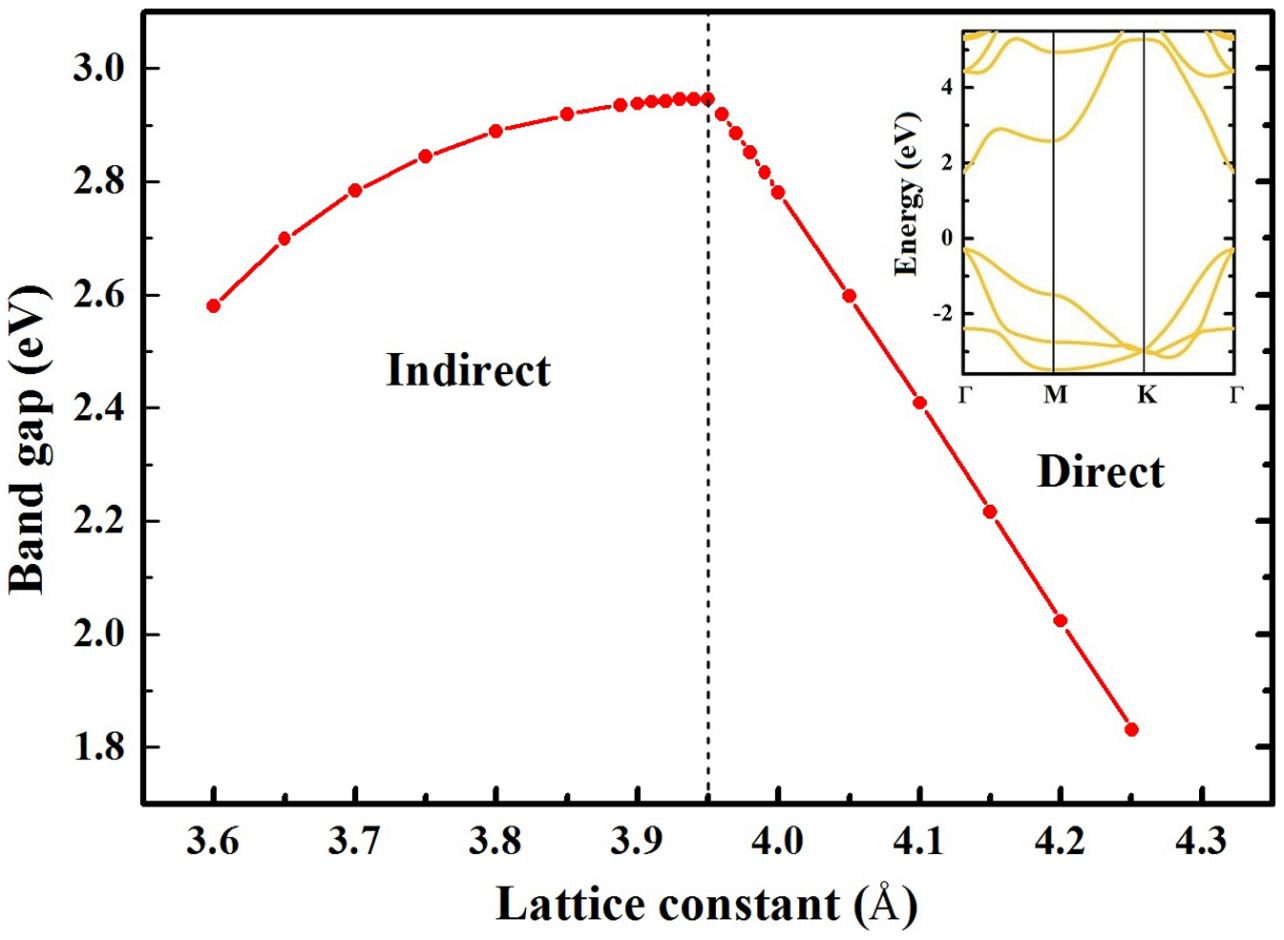
Strain dependence of band gaps for monolayer silicane. Inset indicates the direct band gap of monolayer silicane under tensile stress (a = 4.2 Å).

To understand the indirect-to-direct band gap transition with a band gap reduction of monolayer silicane in SiH/TiO_2_(101) heterojunction, we have studied the influence of strain on the band structure of monolayer silicane. Figure 5 shows that even a slightly increased lattice value of monolayer silicane from the optimum value of *a* = 3.888 Å induces the indirect-to-direct band gap transition and the band gap reduction. The indirect-to-direct band gap transition of monolayer silicane appears when the lattice value is larger than 3.95 Å (1.59%, tensile stress). Furthermore, the band gap of monolayer silicane linearly reduced with the increase of lattice value in the range of 3.95 Å to 4.25 Å. In the case of tensile stress, the increased lattice of *a* = 4.2 Å (8.02%, tensile stress) reduces the band gap of monolayer silicane to 2.023 eV (direct gap at the Γ point), as displayed in the inset of Figure 5. Therefore, the indirect-to-direct band gap transition and the band gap reduction (to 2.082 eV) of monolayer silicane can attribute to the tensile stress of monolayer silicane (6.76% and 7.72% along *x* and *y* directions, respectively) in the SiH/TiO_2_(101) heterojunction.

**Figure 6:**
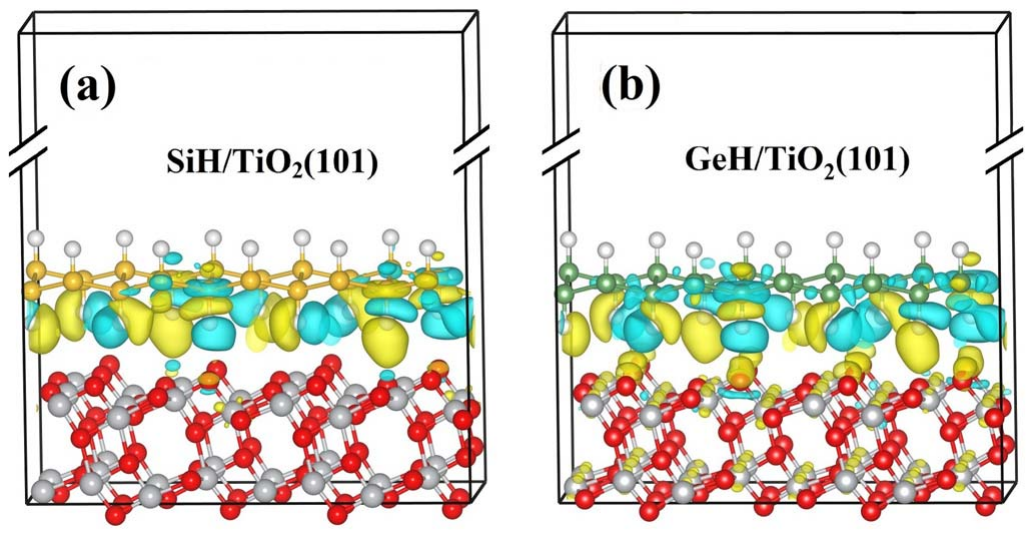
The three-dimensional charge density difference of SiH/TiO_2_(101) and GeH/TiO_2_(101) heterojunctions. (a) SiH/TiO_2_(101) heterojunction. (b) GeH/TiO_2_(101) heterojunction. The yellow region represents charge accumulation and the light blue region indicates charge depletion. The isosurface value is 8.8 × 10^−5^ e/Bohr^3^.

To clarify the charge transfer and separation process, we calculated the three-dimensional charge density difference by subtracting the electronic charges of the monolayer silicane (or germanane) and TiO_2_(101) slab from that of a hybrid SiH/TiO_2_(101) [or GeH/TiO_2_(101)] heterojunction, as shown in Figure 6(a) and Figure 6(b), respectively. It is found that the charge redistribution mainly occurs at the interface region of the SiH/TiO_2_(101) heterojunction, while there is almost no charge transfer from the TiO_2_(101) slab matrix to the interface. The charged interface region of SiH/TiO_2_(101) heterojunction is very similar to the space charge region of p-n junction, in which the electron-hole pair can be effectively separated under the build-in potential. For GeH/TiO_2_(101) heterojunction, the charge accumulation can be found in whole TiO_2_(101) slab, indicating a large amount of charge transfer. These charge accumulation sites may act as recombination centers. To quantify the charge transfer, we performed the Bader analysis^30^ for the charge densities of SiH/TiO_2_(101) and GeH/TiO_2_(101) heterojunctions. The results indicate that there are 0.053 and 0.082 electrons transferred from silicane and germanane to TiO_2_(101) slabs SiH/TiO_2_(101) and GeH/TiO_2_(101) heterojunctions, respectively, which are consistent with the calculation results of binding energies, suggesting that the SiH/TiO_2_(101) heterojunction has more ideal charge redistribution for charge separation, compared to the GeH/TiO_2_(101) heterojunction.

## Discussion

In summary, the electronic properties of silicane (SiH) and germanane (GeH) as photosensitizers on the anatase TiO_2_(101) surface have been studied by using hybrid density functional calculations. The calculated results indicate that the photocatalytic response of anatase TiO_2_ can be improved through monolayer silicane sensitization owing to the fact that the band gap value of silicane (2.082 eV) is suitable for the utilization of solar energy, and its conduction band minimum (CBM) is higher than that of TiO_2_, which lead to an effective photoexcited electron injection from silicane to anatase TiO_2_(101) surface in the SiH/TiO_2_(101) heterojunction. The electronic structures of such a small band gap semiconductor sensitized TiO_2_ have great advantages over these of foreign-element doped TiO_2_ to improve the photocatalytic performance of TiO_2_ in visible-light region. Our results show that the monolayer silicane (SiH) on the anatase TiO_2_(101) surface is a promising TiO_2_-based photocatalyst under visible light. It is expected that this work can motivate experimental scientists to synthesize the designed heterojunctions.

## Methods

The density functional theory (DFT) calculations were performed by using the projector augmented wave (PAW)^31^,^32^ pseudopotentials in the VASP code^33^,^34^. The Perdew-Burke-Ernzerhof (PBE)^35^ parameterization of generalized gradient approximation (GGA) was adopted to describe the exchange and correlation potentials. The cutoff energy of plane-wave basis was set to 500 eV. The Γ point and a Monkhorst-Pack^36^ grid of 1 × 3 × 1 were used for electronic properties calculations and geometry optimizations, respectively. Both the cell parameters and atomic positions were optimized until the force on each ion was smaller than 0.01 eV/Å, and the resulting structures were then used to start the electronic structures calculations. To obtain the correct electronic structures (especially the band gap energies), we used the Heyd-Scuseria-Ernzerhof (HSE06)^28^,^29^ hybrid density functional for energy band structures calculations. In the HSE06 functional, the exchange contribution is divided into short- and long-ranged part. The short-ranged part of PBE exchange is mixed with 25% Hartree-Fock (HF) exchange, and the expression for exchange-correlation in HSE06 is given by: 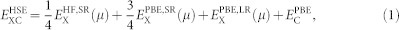
where SR and LR refer to the short- and long-ranged parts of the exchange interaction, and *μ* is the parameter that defines the range-separation of Coulomb kernel. In this work, *μ* = 0.2 Å^−1^ is used.

The binding energies per simulation supercell between the monolayer silicane or germanane and the TiO_2_(101) slabs were calculated by 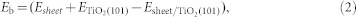
where *E_sheet_*, 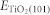, and 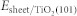 represent the total energies of monolayer silicane or germanane, TiO_2_(101) slab, and the corresponding hybrid systems, respectively. In the systems with weak interface interactions, the weak interactions affect the interface distances and thus influence the energy band structures of the systems. Therefore, the van der Waals (vdW) interactions in SiH/TiO_2_(101) and GeH/TiO_2_(101) heterojunctions have been checked by using DFT-D2 method of Grimme^37^. The differences of total energies and interface distances between PBE and DFT-D2 optimized SiH/TiO_2_(101) and GeH/TiO_2_(101) heterojunctions are less than 5 × 10^-4^ eV and 0.001 Å, respectively, which indicate that it has only negligible influence on the total energies and the stable geometries.

## Author Contributions

D.J.C. and D.P.C. co-produced the original idea; M.N. performed all the DFT calculations. All the authors discussed the results, analyze data and wrote this paper.

## Figures and Tables

**Table 1:** The optimized lattice parameters of SiH/TiO_2_(101) and GeH/TiO_2_(101) heterojunctions; and the absorption properties of monolayer silicane and germanane on anatase TiO_2_(101) slabs

	Lattice parameters (Å)			
	*a*	*b*	E_b_ (eV)	D_z_ (Å)
SiH/ TiO_2_(101)	20.754	7.254	0.092	2.643
GeH/ TiO_2_(101)	21.07	7.312	0.112	2.489
